# Association of myopia with anxiety and mood disorders in adolescents

**DOI:** 10.1038/s41433-024-03170-6

**Published:** 2024-06-13

**Authors:** Itay Nitzan, Or Shmueli, Margarita Safir

**Affiliations:** 1 Department of Ophthalmology, Hadassah-Hebrew University Medical Center, Jerusalem, Israel; 2 Department of Military Medicine, Faculty of Medicine, Hebrew University of Jerusalem, Jerusalem, Israel; 3 Department of Ophthalmology, Shamir Medical Center, Faculty of Medicine, Tel Aviv University, Tel Aviv, Israel

**Keywords:** Paediatrics, Epidemiology

## Introduction

Myopia is a growing global health concern, expected to impact half of the world’s population by 2050 [1]. While research highlights its broad-ranging implications for quality of life, studies focusing on adolescents are limited [2, 3]. Considering the significant influence of mental health on youth well-being, this study aims to explore the association between myopia, anxiety, and mood disorders in a large, nationwide sample of adolescents.

## Methods

This cross-sectional study analyzed data from Israeli adolescents aged 16 to 20 years, evaluated before mandatory military service between 2011 and 2022. Evaluations covered sociodemographic and medical histories, physical examinations, and measurements of refraction and best-corrected visual acuity (BCVA) [4]. The Israeli Defense Forces Medical Corps Institutional Review Board approved the study and waived informed consent due to maintained anonymity.

Myopia was classified using non-cycloplegic right eye spherical equivalent (SEQ) measurements, categorized as mild (−0.75 ≥ SEQ > −3 dioptres [D]), moderate (−3 ≥ SEQ > −6D), and severe (SEQ ≤ −6D) [4]. Diagnoses of anxiety and mood disorders required confirmation by a psychiatric consultant and were categorized according to the ICD-10 [5]. Socioeconomic status was determined by the location of residency at examination [4].

Logistic regression models calculated odds ratios (OR) and 95% confidence intervals (CIs) for anxiety and mood disorders across myopia severity. Multivariable models were adjusted for sociodemographic and anthropometric variables. Additional analyses included stratification by sex, restriction to adolescents with BCVA ≥ 6/9, and those with unimpaired health.

Statistical significance was set at two-sided *P* < .05. Analyses were performed using Statistical Package for the Social Sciences (SPSS) version 29.0 (IBM Corp.).

## Results

Of 891,501 included adolescents (57.7% males; mean [SD] age, 17.2 [0.7] years), 279,419 (31.3%) had myopia: 172,062 (19.3%) mild, 85,310 (9.6%) moderate, and 22,047 (2.5%) severe. Anxiety was diagnosed in 8683 (1.0%) and mood disorders in 4163 (0.5%). Study population characteristics are detailed in Table 1.

**Table 1:** Baseline characteristics of the study population by myopia severity^a^.

	No myopia	Mild myopia	Moderate myopia	Severe myopia	Total
No.	612,082	172,062	85,310	22,047	891,501
Sex					
Male	58.8	53.4	57.1	64.3	57.7
Female	41.2	46.6	42.9	35.7	42.3
Age, mean (SD), years	17.2 (0.7)	17.2 (0.7)	17.3 (0.8)	17.4 (0.8)	17.2 (0.7)
Born in Israel	89.8	90.8	91.7	92.9	90.3
Socioeconomic status					
Low	23.6	26.0	31.3	39.2	25.2
Medium	51.9	51.0	47.6	42.8	51.1
High	24.5	23.0	21.2	18.0	23.7
Complete education	94.4	94.9	93.9	91.3	94.4
BMI, mean (SD), kg/m^2^	22.7 (4.3)	22.8 (4.5)	22.7 (4.4)	22.6 (4.6)	22.7 (4.4)

*BMI* body mass index (calculated by dividing the weight in kilograms (kg) by the square of the height in meters (m^2^)). *SD* standard deviation.

^a^Data presented as column percentage of participants unless otherwise specified.

Adolescents with myopia had higher prevalence of anxiety and mood disorders compared to those without (1.2% vs 0.9% for anxiety; 0.6% vs 0.4% for mood disorders; *P* < .001 for both). Prevalence increased with myopia severity: 1.1%, 1.3%, and 1.6% for anxiety and 0.5%, 0.6%, and 0.7% for mood disorders across mild, moderate, and severe myopia, respectively (*P* < .001 for both).

**Fig. 1:**
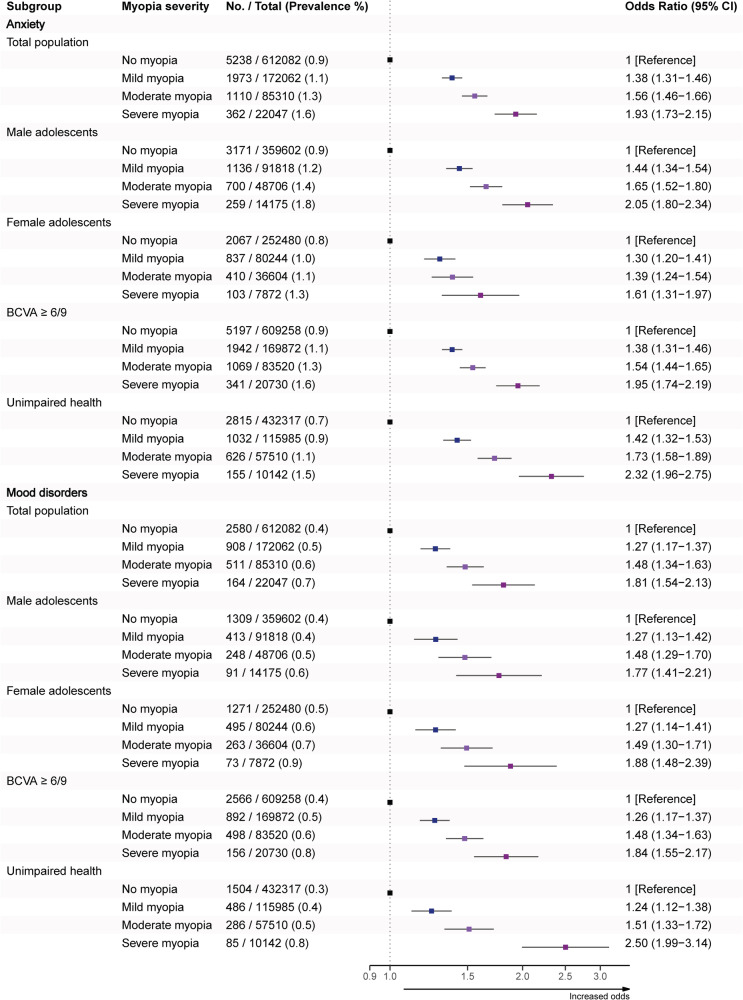
Adjusted odds ratios (ORs) for anxiety and mood disorders by myopia severity. The forest plots present odds ratios and 95% confidence intervals (95% CI) for anxiety and mood disorders by myopia severity. Adolescents without myopia served as the reference group. Models were adjusted for residential socioeconomic status, education level, immigration status, body mass index, and year of evaluation. BCVA best-corrected visual acuity.

Multivariable models confirmed a dose-response relationship between myopia severity and odds of anxiety and mood disorders. ORs for anxiety increased from 1.38 (1.31–1.46) to 1.93 (1.73–2.15) across mild to severe myopia, while ORs for mood disorders rose from 1.27 (1.17–1.37) to 1.81 (1.54–2.13). This association persisted in both sexes, in adolescents with BCVA ≥ 6/9 and those with unimpaired health (Fig. 1).

## Discussion

This study found that adolescents with myopia had up to twofold increased odds of anxiety and mood disorders, regardless of sex, visual acuity, or other comorbidities. The likelihood for both conditions rose with worsening myopia in a dose-dependent manner.

The impact of myopia on quality of life encompasses physical discomfort, limitations in daily activities, and psychosocial effects, especially during the vulnerable period of adolescence [2, 3]. While vision correction measures can alleviate some of these burdens, a gap persists, especially for individuals with high myopia [2, 6]. Our findings underscore the importance of early identification and prevention of myopia progression in children to address both its direct vision-related outcomes and broader psychosocial implications during adolescence.

Limitations of this study include its cross-sectional design, which prevents causality assessment. Additionally, we did not investigate the effects of spectacle wear, contact lens use, and refractive surgery.

In conclusion, our findings indicate a dose-response relationship between the severity of myopia and the prevalence of anxiety and mood disorders among adolescents. This suggests that integrated psychological counselling could be beneficial in the appropriate context as part of myopia management. Future research should focus on developing strategies to mitigate the mental burden of myopia among youth.

## Data Availability

The current dataset is subject to military restrictions; therefore, its availability is limited. The corresponding author will on request detail the restrictions and any conditions under which access to some data may be provided.
